# Building Complexity From Simplicity: A Songbird's Vocal Repertoire Varies Among Populations Despite Similarity of Syllables

**DOI:** 10.1002/ece3.72020

**Published:** 2025-09-10

**Authors:** Aya Marck, Oren Kolodny, Yoni Vortman, Rachel Ben‐Shlomo, Yizhar Lavner

**Affiliations:** ^1^ The Department of Ecology, Evolution and Behavior The Hebrew University of Jerusalem Jerusalem Israel; ^2^ Hula Research Center, Department of Animal Sciences Tel‐Hai College Upper Galilee Israel; ^3^ Department of Biology, Faculty of Natural Sciences University of Haifa – Oranim Tivon Israel; ^4^ Department of Computer Science Tel‐Hai College Upper Galilee Israel

**Keywords:** acoustic variation, dialects, *Pycnonotus xanthopygos*, syllable similarity, vocal repertoire, White Spectacled Bulbul

## Abstract

Vocal communication plays a critical role in understanding animal behavior, evolution, and cognition. We developed an automated system combining audio signal processing and machine learning (supervised and unsupervised) to characterize the vocal repertoire of the White Spectacled Bulbul ( *Pycnonotus xanthopygos* ), a widespread passerine in Israel known for its complex year‐round vocal activity. Analyzing hundreds of field recordings using passive acoustic monitoring (PAM), our system identified thousands of calls, revealing a hierarchically structured vocal repertoire composed of distinct complex vocalizations (*motifs*) and base units (*syllables*). Our results show that different populations possess unique motif repertoires, primarily consisting of population‐specific motifs built from syllables that are similar across genetically distinct populations. This study enhances our understanding of this understudied species and highlights the White Spectacled Bulbul's potential as a model organism for investigating vocal communication and social learning in animals.

## Graphical abstract

How do birds develop their unique vocal repertoires, and how do these vary across populations? Using machine learning and bioacoustics, we analyzed the vocalizations of the White Spectacled Bulbul, revealing population‐specific motifs built from shared syllables. Our findings suggest that while syllable production may be constrained, their arrangement is flexible, shaping distinct local repertoires. This study provides new insights into vocal communication and highlights the potential of automated methods for studying complex animal vocalizations.

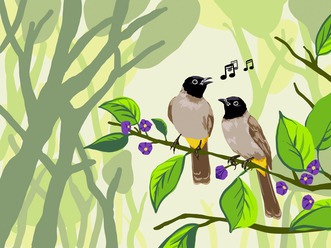

## 1 Introduction

Vocal communication, particularly in social species, has been widely studied across various scientific disciplines, as it provides insights into key biological processes such as cultural evolution, social learning, the origins of language, speciation, and population dynamics. Vocal learning—the ability to acquire and modify acoustic signals through imitation—has been documented in several avian taxa, including passerines, parrots, and hummingbirds, as well as in mammals such as cetaceans, elephants, seals, bats, and primates (Thorpe 1954, 1958; Baptista and Schuchmann 1990; Pepperberg 1994; Janik and Slater 1997; Stoeger et al. 2012; Prat et al. 2015; Oren et al. 2024). Among birds, species that learn their vocalizations tend to have complex vocalizations, with individuals acquiring calls from tutors either early in life, during their first year, or throughout their lifespan (Podos and Warren 2007). These learned vocalizations form the basis of a population's repertoire, which can evolve over time as calls spread among individuals, accumulating modifications through errors in imitation or innovation (Beecher and Brenowitz 2005). This process results in patterns of vocal variation within and between populations. In some cases, these patterns exhibit geographic structuring (Podos and Warren 2007). Species with large repertoires, particularly *open‐ended learners* that continue to acquire new vocalizations throughout their lives, often show high individual variation, making it more difficult to define distinct geographic boundaries in vocal structure (Catchpole and Slater 2003; Lee et al. 2019).

Geographic variation in vocalizations can manifest as either gradual transitions or discrete differences between populations. One well‐documented pattern in geographic acoustic variation is the “clinal” pattern, where vocal differences change progressively across space due to extensive vocal sharing among neighboring individuals (Podos and Warren 2007). This pattern has been widely studied in species with multiple calls in their repertoires, particularly in male song. For example, male Great Tits ( *Parus major* ) possess repertoires of one to six distinct songs, with song sharing decreasing as geographic distance increases, alongside a decline in similarity among shared song elements (Mcgregor and Krebs 1982, 1989). In contrast, some species exhibit a “mosaic” pattern of vocal variation, where populations maintain high within‐group similarity but show distinct differences between neighboring populations. This dialectal structure is typically observed in species with small or single‐song repertoires, such as the Corn Bunting ( *Emberiza calandra* ) and the well‐studied White‐crowned Sparrow ( *Zonotrichia leucophrys* ) (MacDougall‐Shackleton and MacDougall‐Shackleton 2001; Garland and McGregor 2020). In these species, vocal variation is often constrained by a limited number of syllables, which are recombined to produce different vocalizations. While vocal units tend to be shared within and among nearby populations, overall call sharing is significantly higher within populations than between them (Catchpole and Slater 2003). For example, in a study of Marsh Sparrows ( *Melospiza georgiana* ) across six populations, individuals shared calls within their population but had very few shared calls across populations (Lachlan et al. 2018). Similar findings have been reported in Bellbirds ( *Anthornis melanura* ) (Webb et al. 2021), the Rufous‐capped Warbler ( *Basileuterus rufifrons* ) (Demko and Mennill 2019), the Daurian Redstart ( *Phoenicurus auroreus* ) (Lee et al. 2019), and the Black‐capped Chickadee ( *Poecile atricapillus* ) (Baker et al. 2000).

Regardless of repertoire size, vocal differences—both in call types and their acoustic elements—tend to increase with geographic distance. Syllables, however, are often more stable over time and space than entire vocalizations, potentially serving as long‐term markers of population structure (Catchpole and Slater 2003). Despite extensive research on geographic variation, much less is known about how vocal diversity is structured in species with large and complex repertoires and how this variation is distributed across space and between populations. Habitat characteristics play a significant role in shaping the acoustic structure of bird vocalizations. According to the Acoustic Adaptation Hypothesis (AAH), birds are expected to adjust their songs to optimize sound transmission in their specific environments. In densely vegetated habitats, vocalizations typically shift toward lower frequencies (Morton 1975), as low‐frequency sounds propagate with less attenuation compared to high‐frequency sounds (Wiley and Richards 1978). In contrast, species inhabiting more open environments often produce higher‐frequency and broadband calls that are less affected by environmental degradation (Boncoraglio and Saino 2007). Recent studies have further refined this view. For instance, anthropogenic noise can lead birds to raise the pitch of their songs to avoid low‐frequency masking in urban environments (Slabbekoorn and Peet 2003; Winandy et al. 2021). In contrast, body size and phylogenetic constraints have been shown to predict song traits, with no clear association between habitat density and song frequency (Mikula et al. 2021). These findings indicate that while habitat structure does shape the acoustic properties of birdsong, it interacts with additional ecological, morphological, and social pressures.

In recent years, the development of automatic processing tools of vocal communication in animals has opened new possibilities for researchers. Along with the technological progress in machine learning, there are efforts to develop automated algorithms for clustering and classification of basic acoustic units or vocalizations (Ranjard and Ross 2008; Poutaraud 2023). By implementing these algorithms, subjectivity in manual classification and low reproducibility among different researchers are reduced. In addition, it can potentially facilitate the processing of vast amounts of audio data, enabling researchers to obtain a comprehensive picture of vocal behavior of a species, of a habitat's bioacoustic soundscape, or of population differences. Large‐scale bioacoustic studies in the wild are challenging, and in contrast to laboratory experiments, the identity of the individuals and the behavioral context is sometimes missing; the data contain high background noise and irrelevant sections.

**FIGURE 1:**
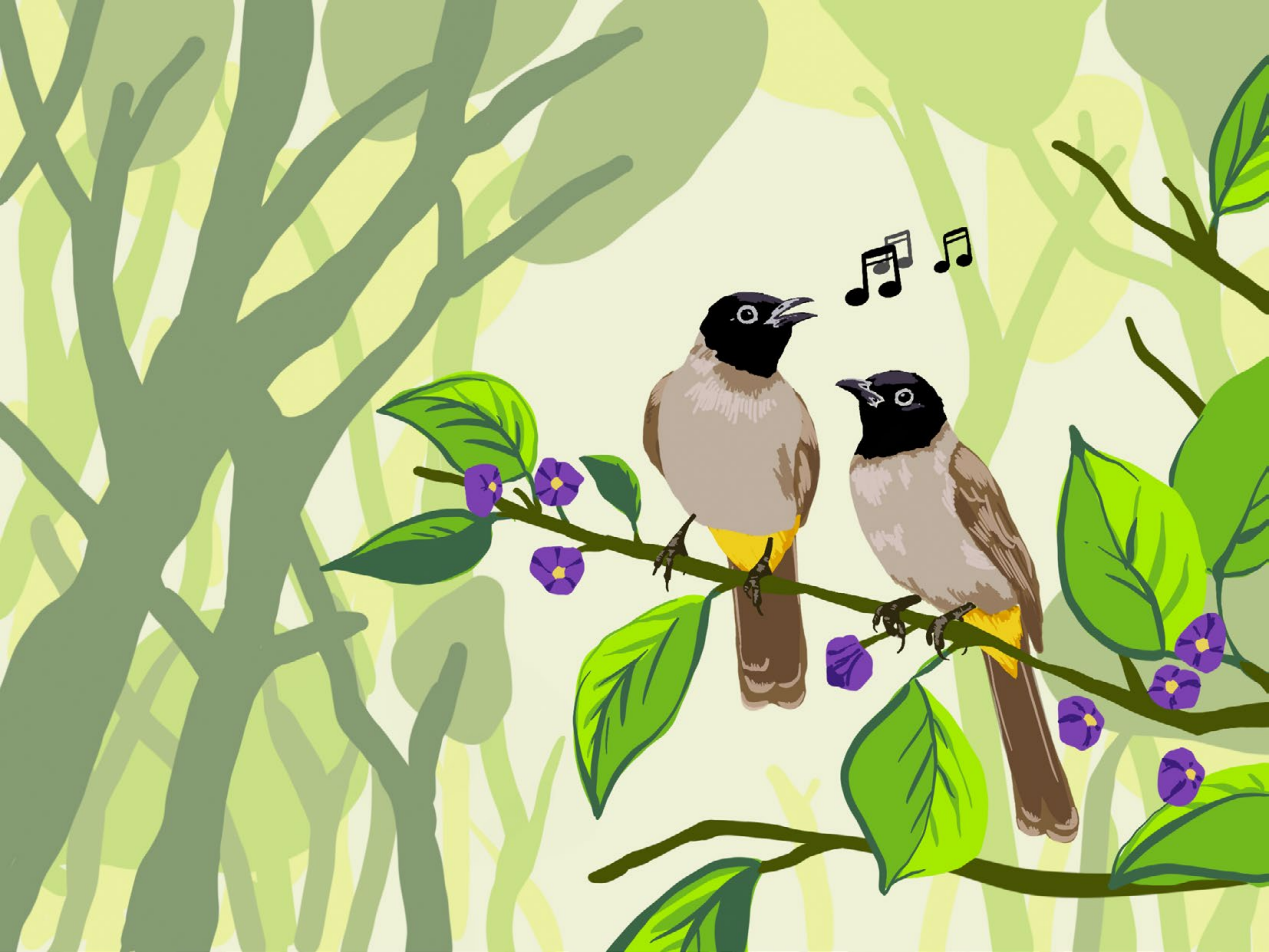
Organism illustration‐the white spectacled Bulbul ( *Pycnonotus xanthopygos* ). Artwork by Aya Marck.

In our study, we addressed several of these challenges by applying deep learning and audio signal processing techniques, which allowed us to efficiently process and analyze large volumes of acoustic data. The data was collected from four populations of the White Spectacled Bulbul ( *Pycnonotus xanthopygos* ), which is a common, widespread passerine in Israel, characterized by tight social bonds between individuals (Hasson 1978) (Figure 1). Beyond its practical advantages—being common, social, and vocal—the white spectacled bulbul exhibits a relatively complex and hierarchical vocal structure that is relatively easy to study in the field. This makes it comparable to species like the Bengalese Finch (Okanoya 2004) and European Starling (Pavlova et al. 2005), which are often studied in syntax research, and sets it apart from classic models like the Zebra Finch or White‐crowned Sparrow, which tend to have simpler, more stereotyped songs (Williams 2004). The Bulbul thus offers a valuable opportunity to study vocal complexity in a natural context. Limited research exists on the behavior of the white spectacled bulbul, particularly regarding its vocal communication. It seems that bulbuls have a relatively limited movement range (without regard to dispersal of young individuals), which forms local and territorial populations (Spiegel and Nathan 2007). This aligns with our observations that bulbuls possess a local vocal repertoire of complex vocalizations, utilized by both sexes throughout the year. This research describes and quantifies the structure of the Bulbul's repertoire, comprising two primary hierarchies of utterances: *syllables* and *motifs*, where a syllable is defined as a base unit of a vocalization surrounded by a pause in singing, and a motif is a complex vocalization that is composed of several syllables (Sainburg et al. 2019). We ask whether syllables and motifs are shared across populations or exhibit population‐specific differences, which may reflect underlying genetic and geographic divergence.

We processed more than 180 h of field recordings, collected at various times of the day and during different seasons, from which we extracted more than 8000 individual Bulbul calls. Utilizing this extensive dataset, which we believe to be a substantial representation of the Bulbul's repertoire, we were able to conduct population‐level analyses, which revealed large differences between the repertoires of different populations but similarities among the base units. We found that the Bulbul uses a limited number of syllables, which might be constrained by anatomical structures or by physiology; thus, they are similar across populations. The specific arrangement of these syllables into motifs, which appears to be flexible, creates distinct repertoires of motifs among close and distant populations.

## 2 General Framework

**FIGURE 2:**
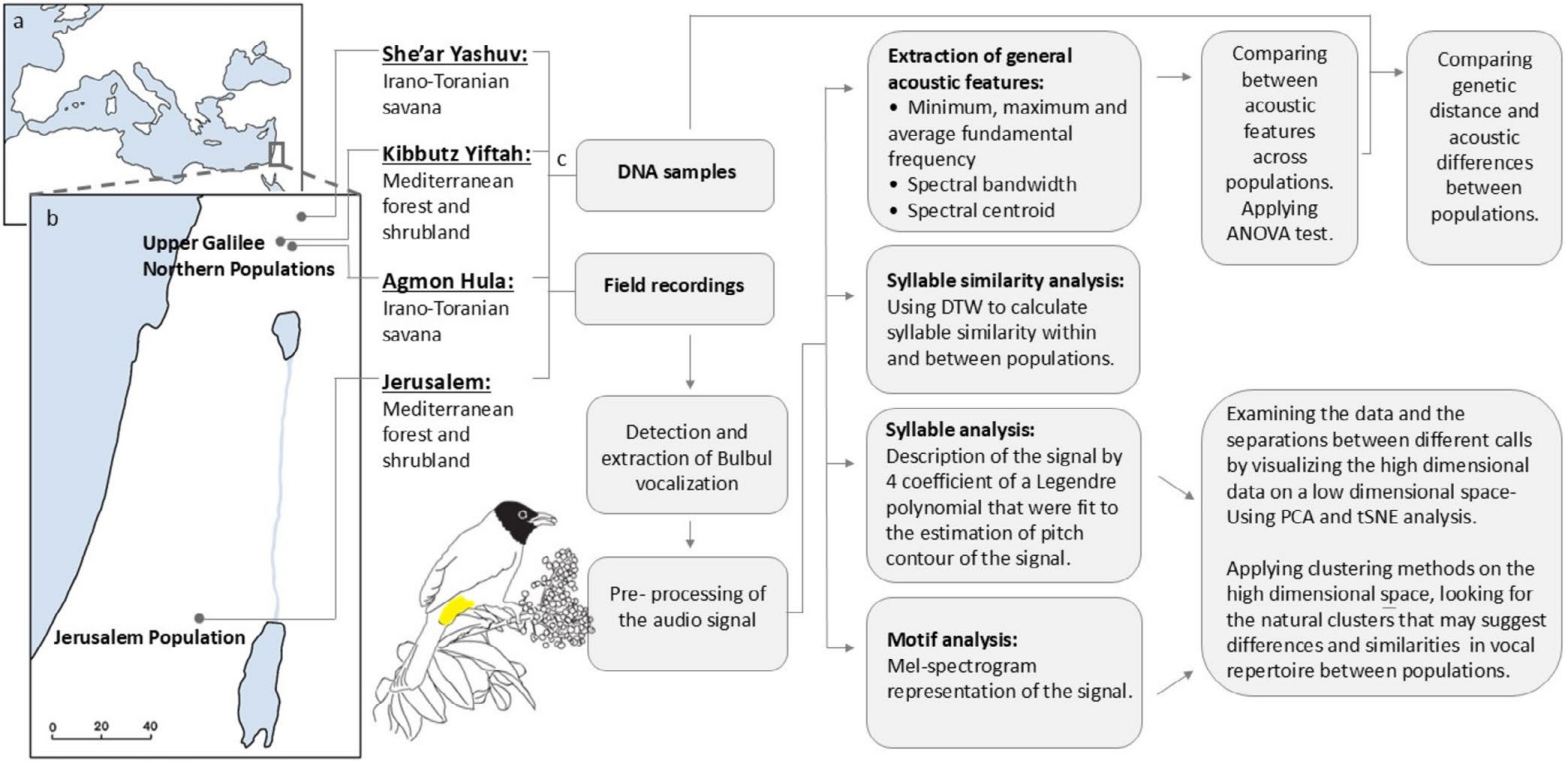
A general scheme of the different methods used to examine population differences and similarities. (a) Map of the medeterenian area. Israel is marked in the east with a gray box. (b) Northern to central Israel with the four populations (gray dots) in which the study was preformed: Three locations in the Hula valley and one in Jerusalem. (c) DNA samples and field recording were taken from all populations.

In this study, several approaches were used to examine and characterize the differences among Bulbul populations (Figure 2). These include: (a) Long‐term acoustic recording in four distinct geographical regions (see acoustic data set, Section 4.1). (b) Analyzing a subset of these field recordings (between March 2021 to August 2021) in order to characterize vocal units (motifs and syllables) and examine similarity and differences between vocal units to define the population's repertoire. (c) Extraction of acoustic features from each population's repertoire to compare general acoustic traits. (d) DNA analysis of a small sample from each population in order to evaluate the genetic distance between the populations and to examine whether the genetic distances are correlated with the acoustic differences. The detection and extraction of the vocalizations, and the various acoustic analysis methods were carried out by an automatic pipeline detailed in Marck et al. (2022).

## 3 Methods

### 3.1 Acoustic Data Set

Our acoustic dataset was collected from four different populations (Figure 2), between March 2021 and August 2021, using eight SM4 automatic recorders (Wildlife Acoustics 2018). We defined four distinct populations based on geographical sites, with each site represented by two recorders placed within the same general area, several hundred meters apart to ensure that more than one individual would be recorded at each population. All recorders worked simultaneously—starting 30 min before sunrise for 2 h, 1 h at noon, and another 2 h during sunset (lasting 30 min after sunset). Six of the recorders were located in Northern Israel, two at each location (She'ar Yashuv, Agmon Hula, and Yiftah), each characterizing different habitat and weather. The remaining two recorders were installed at the bird observatory in Jerusalem, a geographically distinct population being a relative outgroup.

In this study, we analyzed 4 weeks of recordings, taken from March, May, June and August from all four sites that contain more than 180 h of recording (a total of 16 weeks). In order to include vocalizations that are outside the context of courtship (which occur during late spring into summer; April–June), recordings were obtained at various times throughout the year. However, the months selected are those in which Bulbuls tend to be more active compared to the autumn‐winter time. In addition, focal recordings of marked individuals were conducted using Tascam recorder and Sennheiser ME67 long shotgun microphone (detailed in Supplementary S1). These focal recordings were not included in the acoustic analysis, but they were used to assist in training the neural network for call classification.

### 3.2 Pre‐ Processing of the Acoustic Data

The Bulbul vocalizations were extracted from the recordings using BulbulNET; a framework for Bulbul call analysis and identification, which is based on a convolutional neural network (BubulNET, https://github.com/BulbulNET/BulbulNET.git). The framework receives input of raw recordings sampled at 44,100 Hz and returns all detected Bulbul vocalizations as separate audio files. Since the recordings were made in natural environments, the acoustic signal includes various types of background noise, such as vocalizations of other bird species, calls of other animals, human activity, and weather‐related sounds. All of this, together with the distance between the vocalizing bird and the recorder, affects the intensity and quality of the recorded signal. To address these issues, all recordings were pre‐processed using a combination of filtering and masking techniques; the first step was to apply time‐domain bandpass filtering between 700 Hz and 3900 Hz to remove irrelevant frequencies. These cutoff frequencies were chosen because most of the spectral energy of Bulbul vocalizations falls within this range, effectively capturing the majority of calls while minimizing background noise and reducing overlap with vocalizations of other species (Marck et al. 2022). Consequently, ‘median clipping’ and ‘small object removal’ algorithms were applied to the Mel‐spectrogram computed for each segment to reduce background noise (Marck et al. 2022). In addition, since the vocal identification was applied on 0.5 s segments, it did not necessarily begin and end exactly with the motif boundaries. Therefore, we used a simple algorithm that is based on isolated spoken word detection (Rabiner and Sambur 1975) (See Supplementary S2) to achieve an accurate demarcation. The demarcated signal was considered an isolated motif and was extracted for further analysis.

### 3.3 Acoustic Analysis

A total of 8707 motifs were detected and extracted from 4 weeks of recordings from four populations. The vocalizations include various Bulbul calls of different lengths (average: 0.941 s, SD = 0.426 s), each composed of 1–5 consecutive syllables.

For the analysis of the different base units, motifs and syllables, we used two different methods, which were tested and verified in Marck et al. (2022) and shortly detailed here. These analyses were conducted using the Python 3.8 environment (with Librosa library 0.9.1 functions for the audio analysis):

1. MFB for motif analysis‐a mel‐spectrogram was calculated for each motif signal, with 35 mel filters, using a frame length of 512 samples and a hop length of 128 samples. Consequently, a simple image processing technique (median clipping) was applied to increase the SNR. Finally, one feature vector which represents the complete vocalization is computed (Figure S3a,b).
2. Extraction of the fundamental frequency contour for syllable analysis‐ the fundamental frequency contour $f_{0}\left(t\right)$ was estimated using zero‐crossing rate analysis applied on a bandpass signal with the relevant frequencies (700–3900 Hz). This estimation was verified using the YIN algorithm (De Cheveigné and Kawahara 2002). For each syllable, a Legendre polynomial was fitted to model $f_{0}\left(t\right)$, and the polynomials coefficients were used as a feature vector (Figure S3c).

Based on these analyses, a feature vector was generated for each vocal unit (mel‐spectrograms for motifs, and polynomial coefficients for syllables), enabling further comparisons of the populations, as follows:

#### 3.3.1 Extracting Acoustic Properties From Motifs

Five acoustic features were extracted from the 8707 motifs collected to examine acoustic differences between the populations. The features were extracted by initially computing a clean mel‐spectrogram of the motif signal and identifying syllable boundaries within the motif based on spectral energy between 700 and 3900 Hz. Subsequently, a new signal of concatenated syllables was created (by removing the intervals between syllables), and finally, the fundamental frequency contour was computed (using two algorithms‐ ZCR and PYIN) in order to extract frequency parameters (maximum, minimum, average fundamental frequency, bandwidth and spectral centroid) (Figure S4).

#### 3.3.2 Defining Local Repertoire by Clustering Methods

In our previous work, we demonstrated that dimensionality reduction and clustering methods can automatically identify vocalization groups without prior labeling. Moreover, these methods enhance reproducibility and can uncover patterns that might otherwise go unnoticed. Building on this approach, we applied a similar workflow in this study to examine differences and similarities in vocal units within and across populations. We applied *K*‐means clustering to data reduced via principal component analysis (PCA), reducing 1190 dimensions extracted from the signal spectrogram to 10. Dimensionality reduction not only helped eliminate noise and irrelevant features but also clarified the data's central tendencies, facilitating group separation. To ensure cluster homogeneity, we performed a manual examination by randomly selecting 30 vocalizations from each cluster and another set of 30 closest to each cluster's centroid. These were evaluated through visual and auditory inspection. For visualization, we used t‐distributed stochastic neighbor embedding (t‐SNE), which generally preserves the global structure of the data in two dimensions.

The optimal number of clusters for the *K*‐means analysis was determined by silhouette score, which measures how close each point in a cluster is to other points in the same cluster compared to other clusters, with values in the range [−1, +1], where high values indicate well‐separated and distinct clusters and elbow method. Furthermore, to measure how much motifs and syllables are shared or differ among populations, homogeneity score was calculated as follows:

$$h=1-\frac{H\;\left(C\right|K)}{H\left(C\right)}\;\text{where}\;H\;\left(C\right|K)=-\sum_{c,k}\frac{n_{\mathrm{ck}}}{N}\log\left(\frac{n_{\mathrm{ck}}}{n_{k}}\right)$$

The score is defined using Shannon's entropy, whereas *H*(*C*|*K*) is the conditional entropy of the class distribution given the cluster prediction and represents the ratio between the number of samples labeled *C* in cluster *K* and the total number of samples in cluster *K* (Rosenberg and Hirschberg 2007). The score measures the extent to which data in a cluster (motif or syllable) contain members of the same class (from the same population, in our case), and is bounded between 0 and 1, with low values indicating low homogeneity.

#### 3.3.3 Syllable Similarity Analysis

Syllables were extracted from various motifs across all locations to measure similarity within and between populations. This analysis was restricted to clear signals due to the brevity and often indistinct nature of some of the syllables. Therefore, syllables were manually labeled and classified according to three criteria—frequency range (bandpass), number of inflections at the fundamental frequency, and position within the motif. As syllables differ in duration, it is necessary to calculate the similarity (or the distance) between signals from each pair of syllables after they have been time‐aligned using dynamic time warping (DTW) (Gold et al. 2011).

**FIGURE 3:**
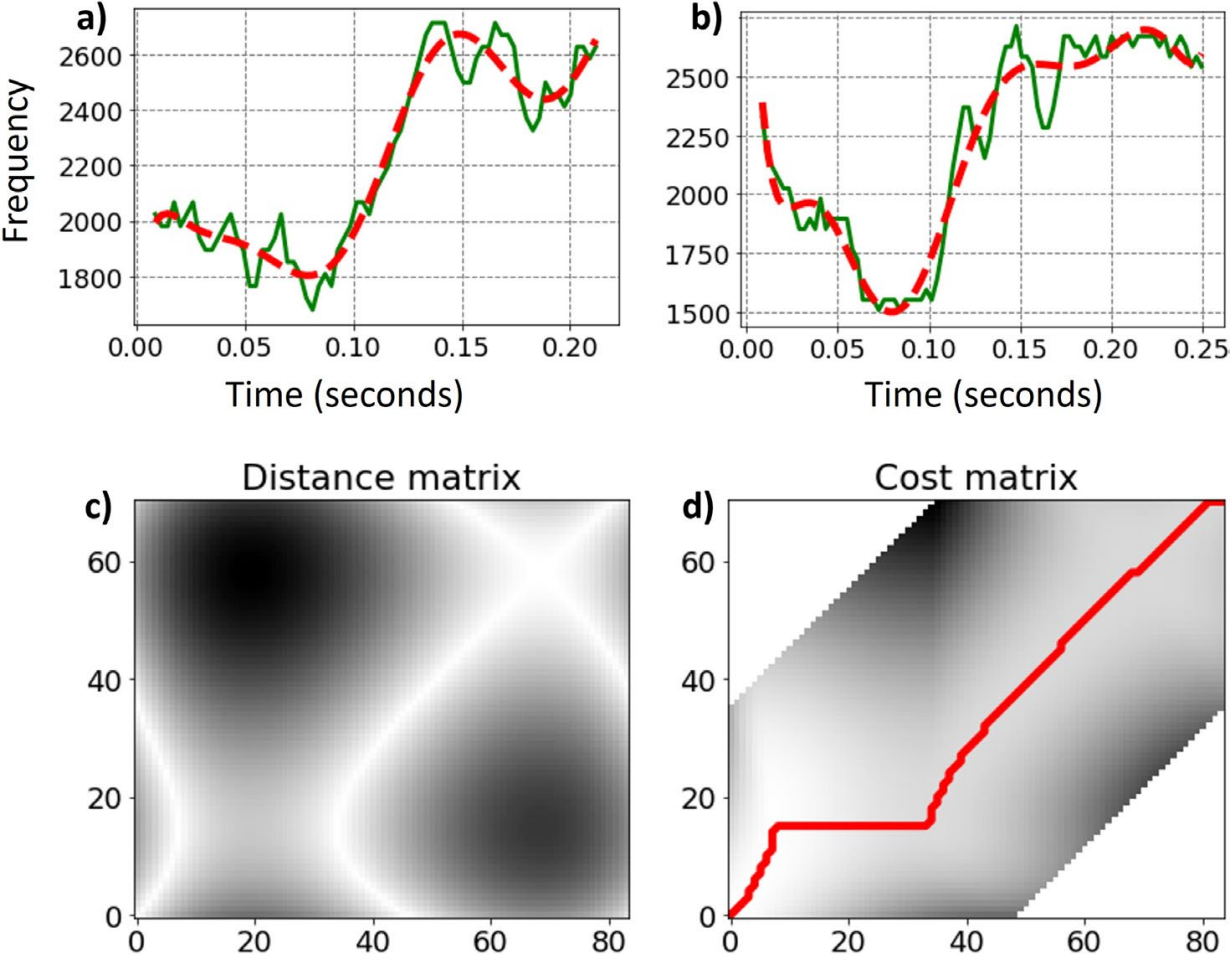
Example of two “tuyu” syllable fundamental frequency contour (green) and their corresponding Legendre polynomial fit (dashed line, red), recorded in Jerusalem site (a) and Agmon site (b). An example of these two syllables' distance matrix after alignment using DTW (c), The optimal path to calculate the minimal cost using a Sakoe‐Chiba constraint (d). The similarity score that was obtained was 0.289 (values closer to 0 indicate high similarity and values that are closer to 1 indicate low similarity).

The dataset for this analysis included 1317 examples of different syllables, excerpted manually from 405 recordings of 27 distinct motifs from all locations. Bulbul calls are mostly built up from discrete units that are easy to demarcate as syllables based on a gap on either side. Therefore, the vocalizations were segmented into syllables by visually inspecting the spectrogram and setting syllable start/end points. Each syllable was represented by a mel‐spectrogram that was computed using a frame length of 512 samples (11.6 msec. with fs = 44,100) and a hop length of 64 samples (1.5 msec.). A total of 50 mel filters were used, with a maximum frequency of 3900 Hz. Consequently, a distance matrix was computed for each pair of syllables in the dataset, using a cosine distance between the columns of the mel‐spectrograms as follows:

$$d{\left(i,j\right)}_{X,Y}=1-\frac{\left\langle X\left(i\right),Y\left(j\right)\right\rangle }{\left\|X\left(i\right)\right\|\left\|Y\left(j\right)\right\|}$$

where $X\left(i\right)$ and *Y*(*j*) are the *i*‐th and *j*‐th frames of the syllables, represented by the corresponding columns of the mel‐spectrograms of the syllables *X* and *Y*, respectively, and $\left\langle X\left(i\right),Y\left(j\right)\right\rangle$ is the inner product between $X\left(i\right)$ and *Y*(*j*). Finally, the distance between the syllables in each pair was computed after alignment using DTW with a Sakoe‐Chiba constraint (Kamper 2017) with a radius of 0.35 (Figure 3), and was defined as the minimal cost along the optimal path divided by the total number of bins for both syllables:

$$\text{dist}\left(X,Y\right)=\frac{\min\left(\text{cost}\left(D_{X,Y}\right)\right)}{N_{X}+N_{Y}}$$

Where $D_{X,Y}$ is the cumulative distortion matrix of the two syllables (Gold et al. 2011):

$$D_{X,Y}\left(i,j\right)=d{\left(i,j\right)}_{X,Y}+\min\left\{\begin{array}{c} D_{X,Y}\left(i-1,j\right)\\ D_{X,Y}\left(i,j-1\right)\\ D_{X,Y}\left(i-1,j-1\right) \end{array}\right.$$

This analysis was also performed using the fundamental frequency contour as the syllable representation, yielding similar results.

### 3.4 DNA Sampling, Extracting and Amplification

DNA samples from feathers and blood were taken from 80 individuals that were captured using mist nets at the four locations listed above during the summer of 2020. Blood was collected from the brachial vein, and feathers were collected from the bird's ventral plumage. DNA was extracted using the DNeasy Blood and Tissue Kit (Qiagen) according to the manufacturer's protocol. A total of 65 (23 from the Agmon, 17 from Jerusalem, 13 from Shear yashuv and 12 from Yiftah) DNA samples were successfully extracted. Polymerase chain reaction (PCR) amplification was used to determine sex and to allow population genetic analysis using microsatellite markers. Sex was determined following Griffiths et al. (1998), using the P2/P8 primers. Since there are no known microsatellite markers of the white spectacled bulbul, we amplified 12 microsatellite loci reported on related species (Lokugalappatti et al. 2008; Wu et al. 2011; Page et al. 2014; Dawson et al. 2000; Table S5). Eventually, five polymorphic microsatellite loci were selected: Heca8, Pf135, Pf151, Pf152, and Pf177. The selected loci were amplified with appropriate sets of primers (Table S5); one primer of each set was 5′ labeled with different fluorescent dyes. 1 μL of each PCR product with internal standard (GeneScan 500 LIZ dye Size Standard, Applied Biosystems) was run on the 3730xl DNA Analyzer, Applied Biosystems. Microsatellites' allele identification and genotyping were determined directly from the chromatographs using Peak Scanner Software, Applied Biosystems.

### 3.5 Genetic Distance

Allele frequencies were calculated from five microsatellite loci in order to examine the genetic distance between populations (Eight loci out of 12 potential loci were successfully amplified, including one monomorphic locus, and two Z‐linked loci that are suitable mainly for analysis of males, therefore these loci were excluded from the analysis). The genetic distance between the four populations and the average number of alleles per locus is presented in (Tables S6 and S7). Genetic data was analyzed using GenAlex software, version 6.2 (Peakall and Smouse 2006). We examined allele frequencies, expected and observed heterozygosity, Nei's genetic distance, *F‐*statistics (Fixation Index) and deviation from Hardy–Weinberg equilibrium.

### 3.6 Statistical Analysis

To assess differences between syllable groups, we first tested the assumptions for parametric tests. The data did not meet the assumption of normality, as indicated by the Shapiro–Wilk test (*p* < 0.05), and Levene's test revealed unequal variances (*p* < 0.05). We assume that the samples are independent, based on the study design and sampling method. Therefore, we applied the Kruskal–Wallis test, a non‐parametric alternative to ANOVA, followed by Dunn's post hoc test to compare differences between groups. The same approach was used for the acoustic feature analysis, where the normality test also indicated significant deviations from normality (*p* < 0.05) and Levene's test showed unequal variances (*p* < 0.05). Consequently, the Kruskal–Wallis test was applied, followed by Dunn's post hoc test to compare differences between populations. As the data is highly dimensional, we applied the non‐parametric PERMANOVA test to evaluate the differences between groups (Anderson 2001; Anderson and Walsh 2013; Supplementary S8).

To examine correlations between genetic, geographic, and acoustic distance matrices, we used the Mantel test, which evaluates the relationship between pairwise distance matrices of equal dimensions.

All statistical analyses were conducted in Python 3.8, using the scipy, sklearn, and statsmodels packages.

## 4 Results

### 4.1 Differences in Vocal Repertoire Between Populations

**FIGURE 4:**
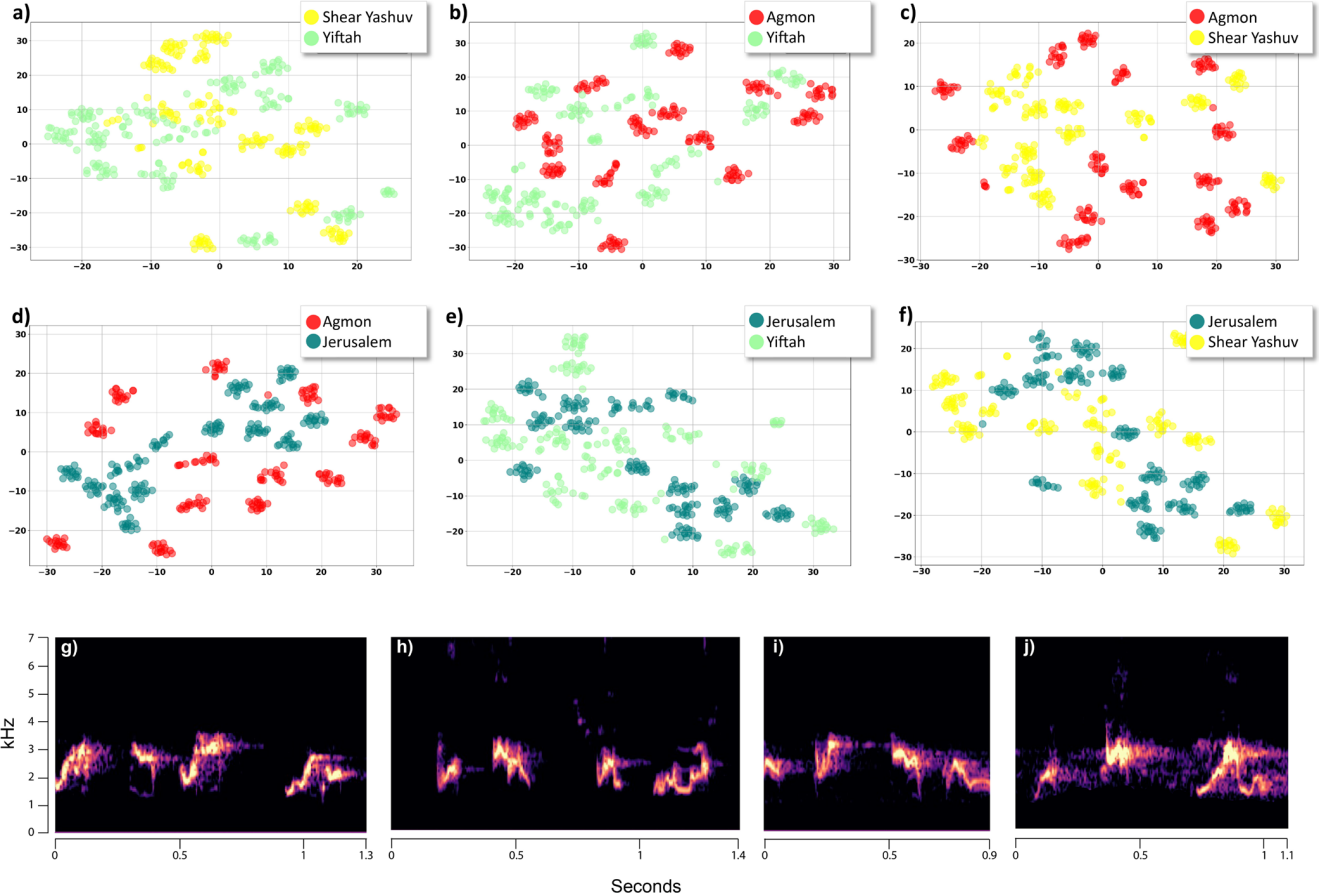
(a–f) A comparison between motif repertoires of different populations visualized on a two‐dimension space using t‐SNE. For the visual representation of motifs and syllables we choose only clear signals. For each pair of populations, a selection of 30 motifs closest to their corresponding centroid from each cluster was included. This selection was based on the clustering analysis done using *K*‐means on a PCA multi‐dimenational space. In most cases, motifs from one population do not overlap or merge with motifs from a second population. For example, Figure 4d presents the Agmon motifs (red points) and Jerusalem motifs (green points) where each color is divided into small groups (different motifs) and they barely overlap with groups from the other color. (g–j) – examples of motifs' spectrograms from each population‐ (g) Agmon, (h) Jerusalem, (i) She'ar Yashuv, (j) Yiftah.

Bulbul motifs are clearly separated by silent intervals and typically consist of 3–6 syllables (Figure 4g–j), although single syllables can also occur in isolation. Individuals may repeat the same motif in sequence or alternate between several different motifs. As a territorial species, bulbuls vocalize year‐round, with a noticeable increase during the courtship season. Each site may host dozens of individuals occupying sub‐territories, and individuals within the same area often appear to share the same set of motifs. The recordings capture a broad variety of motifs and individuals, as they span different times of day and season, and include data from two recorders placed within the same area. While some motifs may be repeated by the same individual on the same day, field observations confirm that multiple individuals are active and vocal within each population.

**FIGURE 5:**
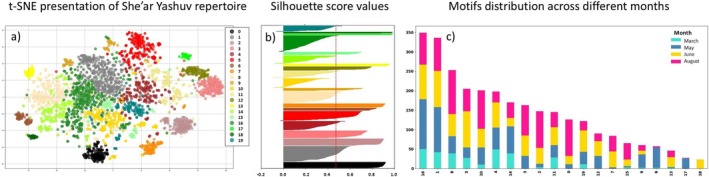
An example of She'ar Yashuv population repertoire. (a) A visualization of She'ar Yashuv Bulbul motifs repertoire (2867 calls) using t‐SNE. Each color represents a cluster of motifs. The number of clusters was estimated using both Elbow method and Silhouette score. The clusters were verified by visual and audio inspection. Motifs 1 and 16 which are the most commonly used and are present in all weeks (c), are distributed in the middle. Motifs 0, 7, 17 and 18 each form a distinct cluster, were cluster 17 and 18 are found exclusively in 1 week (the color represent the same cluster in figures a and b). (b) Silhouette score displaying the clustering quality of the data when *k* = 20. The red line is the average silhouette score for all clusters‐ 0.453 (The highest score that was obtained). (c) She'ar Yashuv motif clusters presented with monthly distribution. The horizontal axis represents the different motif clusters‐corresponding the numbers on (a), and the vertical axis represents the number of identified calls. Each color represents a different week in the year‐ from March, May, June and August.

In a preliminary study at the Yiftah site, we collected focal recordings over several months using a directional microphone. Auditory and spectrographic inspection revealed at least 25 distinct motifs, an estimate that is closely matched with the motif clusters identified through t‐SNE and clustering analyses, which grouped similar motifs without manual labeling. Comparable results were found at other sites, using thousands of vocalizations extracted from different times of day and different seasons, with 18 to 25 distinct motifs clusters per population (Figure 5), representing a particularly large repertoire (Leighton and Birmingham 2021). The clusters were found by a process of unsupervised learning during which we used Principal Component Analysis (PCA) to reduce the dimensionality of the data, followed by clustering with *K*‐means. The number of classes was set using both the highest silhouette score and elbow method (Figure 5b). This process is detailed in Methods (see Section 3.3.2) and was tested on bulbul calls in Marck et al. (2022). A comparison of the populations' repertoires, visualized using t‐SNE in a two‐dimensional space, revealed that most motifs differ between populations, with the exception of a few that are similar and appeared to overlap with other populations' motifs (Figure 4). This process was validated through auditory and visual examination of the spectrograms, confirming that differences between motifs from different populations are distinguishable.

In order to measure the degree of overlap in motifs and syllables between populations, we calculated a homogeneity score which measures the extent to which data in a cluster belong to the same population. This score ranges from 0 to 1, with lower values indicating less homogeneity (see Section 3.3.2). The score was applied to the clusters produced by *K*‐means. Our results show that motifs are relatively homogeneous within populations (0.874), whereas syllables are extensively shared between populations, with few syllables being population‐specific (0.252). These patterns are clearly illustrated in the t‐SNE visualizations (Figures 4 for motifs and 6 for syllables). We also applied PERMANOVA to compare between clusters of populations' repertoires and found a significant difference between groups (*F* = 0.046, *p* = 0.0002) (Supplementary S8).

### 4.2 Shared Syllables Among Populations

**FIGURE 6:**
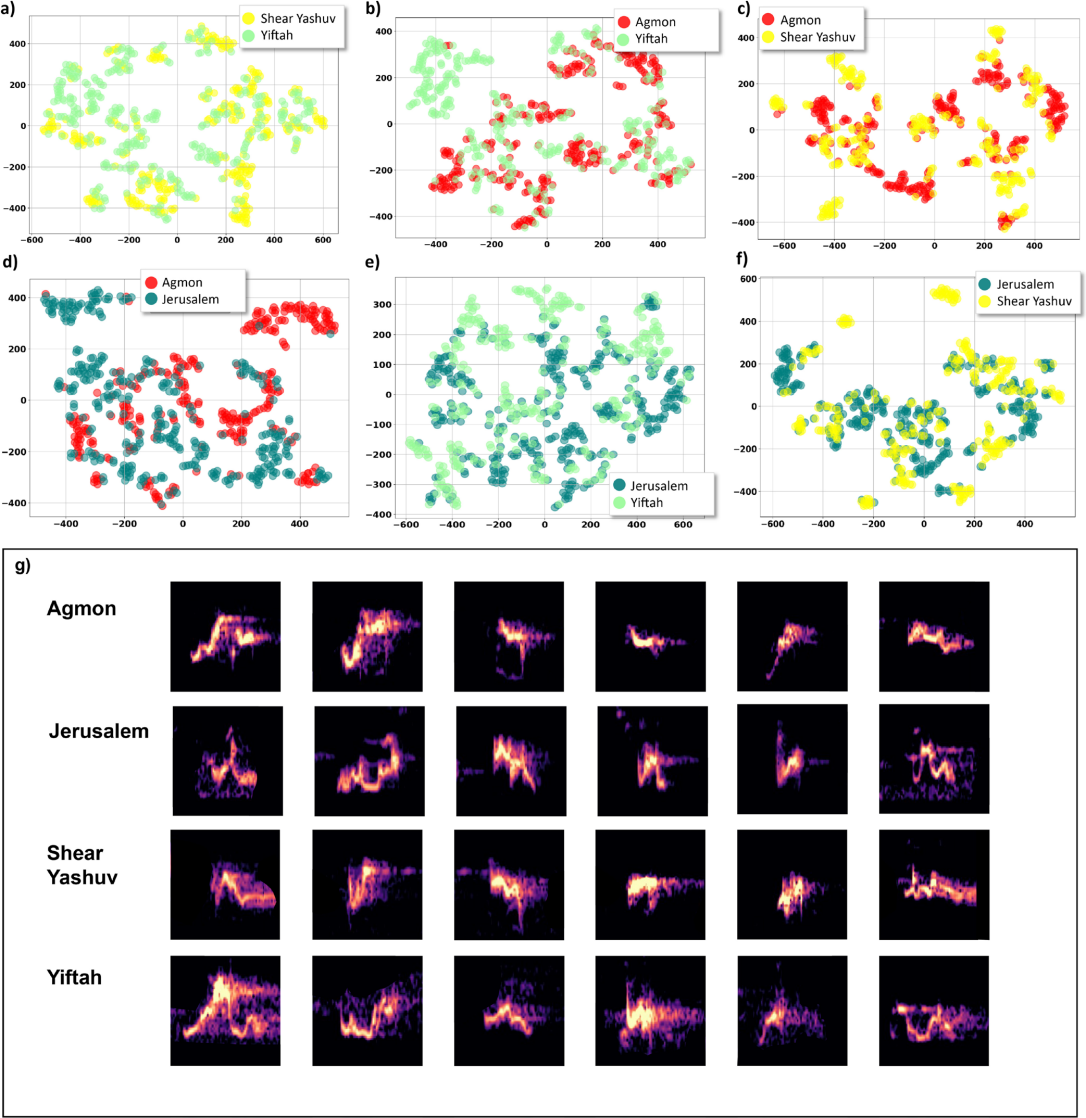
(a–f) A comparison of syllables between populations presented on a two‐dimensional plane using t‐SNE. Each sub‐figure depicts a pair of populations, each containig a set of manually extracted syllables. The figure clearly shows the overlapping between clusters of syllables of populations from different locations, though some clusters are separate. For instance, Figure 6d present syllables extracted from Agmon motifs (red points) and syllables from Jerusalem motifs (green points), with some syllables seemingly separated (upper right and upper left corners) and others overlapping, suggesting to be shared between populations. (g) – examples of syllables spectrograms from each population.

Visual inspection of the spectrogram, combined with listening to vocalizations, suggests that the syllables composing motifs appear similar across different populations. To validate this observation, a total of 1317 syllables were extracted from clear recordings of commonly occurring motifs. A visualization of the data using t‐SNE (Figure 6) revealed substantial overlap in syllables across different populations, in contrast to the distinct motif patterns observed (Figure 4). This suggests that different motifs may be constructed from similar base units (Figure 6).

In addition, we hypothesized that syllables identified as “same” based on subjective auditory inspection would exhibit high similarity, while “different” syllables, both within and between populations, would show lower similarity. To test this, we quantified syllable similarity across populations using Dynamic Time Warping (DTW) (see Section 3.3.3) and found that the “same” syllables from different locations were significantly (*p* < 0.001, Kruskal–Wallis test followed by Dunn's post hoc test) more similar to each other than “different” syllables from the same population (mean similarity score of 0.231, SD = 0.006 and 0.398, SD = 0.004, respectively, with lower scores indicating higher similarity) (Figure S9).

### 4.3 Differences in General Acoustic Features Among Populations Reflect Genetic Distance

**FIGURE 7:**
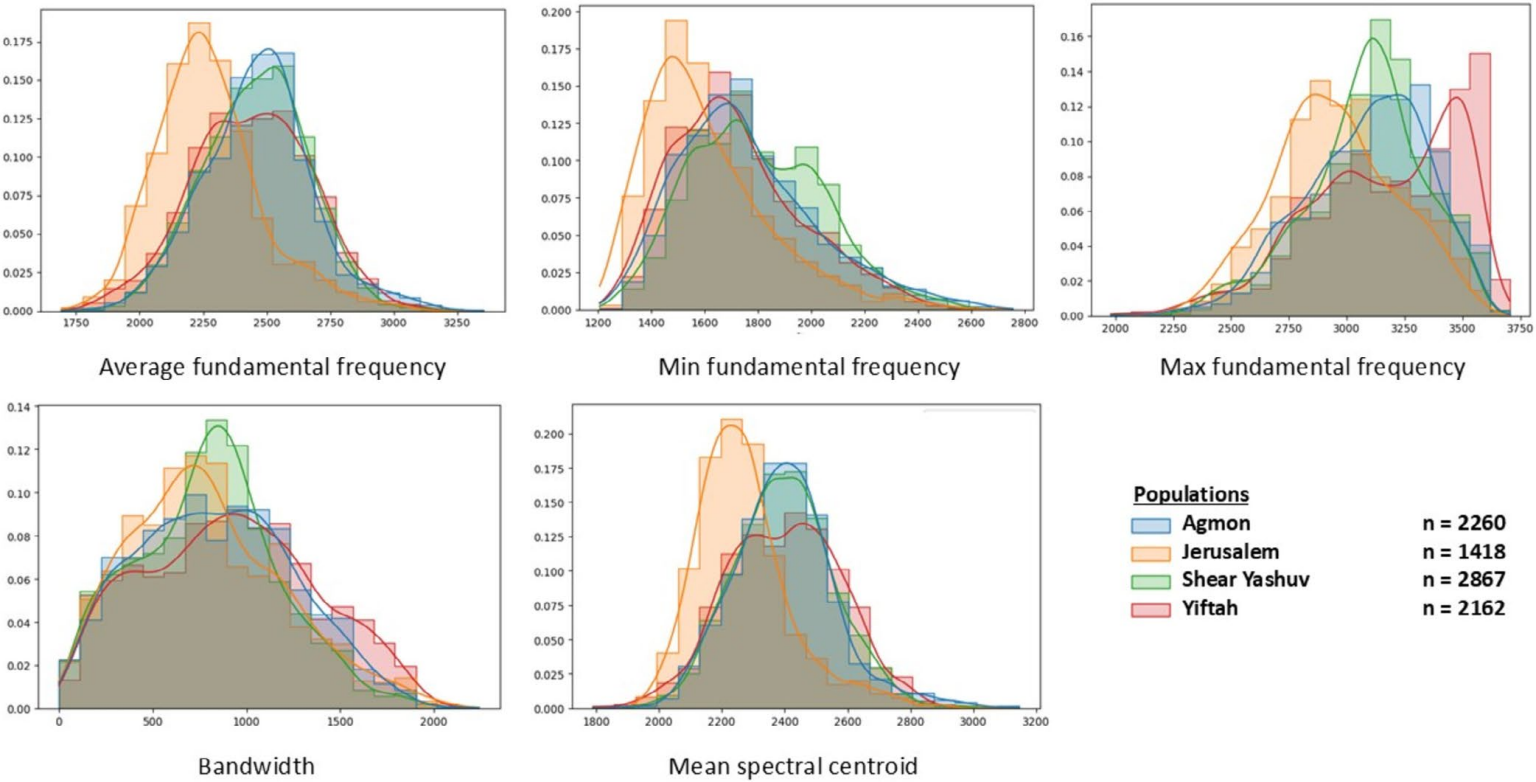
Histograms of five basic acoustic features (minimum, maximum and average of the fundamental frequency, bandwidth, and spectral centroid) that were extracted from motifs of each population repertoire. Different populations are represented in different colors. The Jerusalem population (orange color) is significantly different from all other groups in four out of the five features (*p* < 0.001), with generally lower frequencies.

Acoustic properties may vary between populations depending on body size, use of different vocalizations, genetic differences, and other factors. We analyzed all vocalizations that were detected using BulbulNET (a total of 8707 motifs) and compared five basic acoustic features among populations (minimum, maximum and average of the fundamental frequency, bandwidth and spectral centroid). Using the Kruskal–Wallis test and post hoc test, we found that the Jerusalem population is significantly different from all other groups in four out of the five features (*p* < 0.001), with generally lower frequencies (Figure 7, detailed in Table S10). For example, in Jerusalem, the average fundamental frequency as well as the minimum and maximum fundamental frequency are significantly lower than those in the other locations.

**FIGURE 8:**
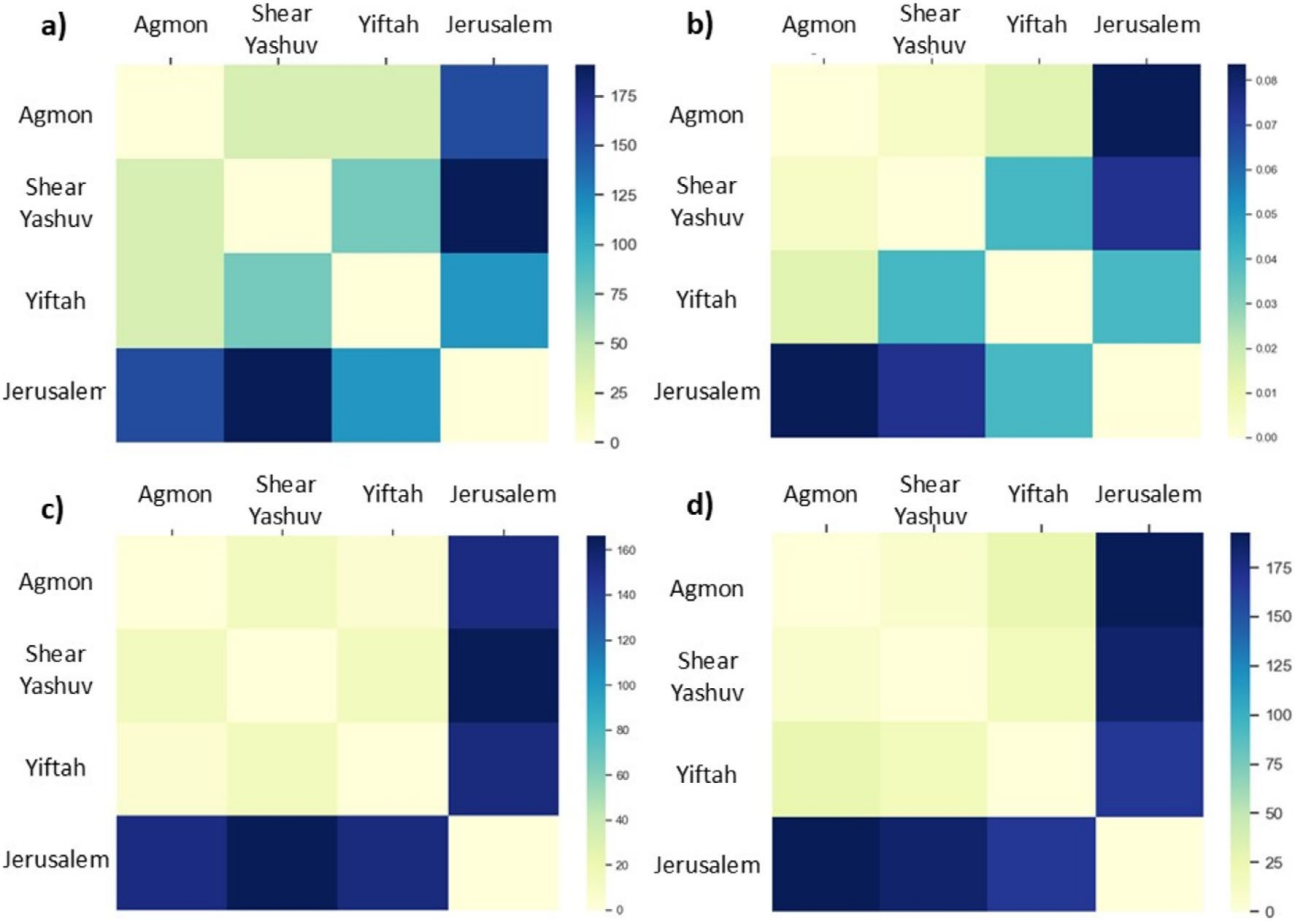
Distance matrices of (a) Acoustic feature—minimum frequency, (b) Genetic distance, (c) Geographic distance and (d) Acoustic feature—average frequency. There is a high correlation coefficient between genetic distance and the average fundamental frequency (0.850, *p* = 0.042), and between genetic distance and the minimum frequency (0.934, *p* = 0.042). When comparing other features with genetic distance (e.g., Maximum frequency and spectral centroid) the correlation coefficient is not significant (0.588, *p* = 0.166; 0.806, *p* = 0.125 respectively).

In addition, the Bulbul population in Yiftah uses a wider range of frequencies (a wider average bandwidth) compared to all other populations. When comparing genetic distance matrices to acoustic features matrices using the Mantel test, we found a significant correlation coefficient between genetic distance and the average fundamental frequency (*r* = 0.85, *p* < 0.05) and between genetic distance and the minimum frequency (*r* = 0.934, *p* < 0.05) (Figure 8). Overall, the Jerusalem population is acoustically, geographically, and genetically furthest from the northern populations (Figure 8). Among the northern populations, despite greater geographic distance between She'ar Yashuv and the Agmon (14 km) than Yiftah and the Agmon (6 km), the genetic distance and some of the acoustic features are much minor (Table of genetic distance is detailed in Supplementary S6).

We found no correlation between the genetic distance and the geographic distance among northern populations (Mantel test *r* = 0.335, *p* = 0.5).

## 5 Discussion

### 5.1 Vocal Differences Between Populations

Geographic variation between repertoires of populations has been demonstrated previously in many bird species (MacDougall‐Shackleton and MacDougall‐Shackleton 2001; Podos and Warren 2007; Lee et al. 2019; Garland and McGregor 2020), typically following one of two patterns: gradual change or distinct differences between individuals or populations (Podos and Warren 2007; Catchpole and Slater 2003). In recent years, there has been an increasing use of advanced signal processing and machine learning techniques for processing and analysis of bioacoustic signals (Hao et al. 2022; Stowell 2022) incorporating multiple parameters and enabling new discoveries on a larger scale. These techniques include deep autoencoders (Kohlsdorf et al. 2020), deep convolutional neural networks (CNN) (Stowell 2022), similarity measures with alignment using DTW (Tan et al. 2015), and others. Following a similar approach, we used machine learning‐based signal processing to analyze the vocalizations of four Bulbul populations in Israel and revealed a particularly large vocal repertoire of complex sounds comprised of base units. Moreover, while the vocal repertoire significantly differed between genetically and geographically distinct populations, similar base units are shared. In the four Bulbul populations examined, each population exhibits a unique vocabulary consisting of 18–25 distinct motifs (complex sounds) that repeat themselves (Figure 4). We observed significant differences between repertoires using two different approaches: (1) comparing basic acoustic features (frequency characteristics) and (2) analyzing the spectrograms, which contain multiple parameters (e.g., processing the time‐frequency representation of the signal). The first approach, that is, comparing basic acoustic features between populations, highlights distinct differences between the Jerusalem population and the northern populations and is mainly expressed in lower frequencies of the Jerusalem repertoire (Figure 7). This population is also genetically different, and therefore it is possible that some of the differences in the basic acoustic properties are the result of variations between populations' morphology. Differences in body size, in the shape of the beak, or the syrinx are genetically determined and are expressed in acoustic variation (Bradbury and Vehrencamp 1998; Mikula et al. 2021). Habitat‐specific selection pressures may contribute to the observed acoustic differences between populations, as environmental characteristics are known to influence the acoustic structure of bird vocalizations. Among the populations in the north, there is no clear correlation between the geographical distance, the genetic structure, and acoustic characteristic similarity. We assume that the northern populations are separate from each other, as studies suggest a relatively small home range, though dispersal may occur. The Agmon and She'ar‐Yashuv populations share similar acoustic properties and genetic structure despite the geographic distance (14 km) between them. This is probably due to connectivity between these two populations, as both are within the Hula Valley, which is characterized by open Irano‐Turanian savanna vegetation and agricultural fields. The population in Yiftah was found to be genetically distant and acoustically different, despite the short distance from the Agmon population (5 km). This may be explained by the different habitats or the possible ecological barrier between the Hula Valley (where the Agmon and She'ar Yashuv are located) and the Ramim mountain range (where the Yiftah population is located) which is surrounded by a Mediterranean forest and is 400 m higher. The second approach, that is, analyzing the spectrograms, highlights the differences between populations' repertoires, as we found different motifs in each population. This suggests that plastic behavior and social learning are major factors in the bulbul's vocal communication. The bulbul's wide and diverse repertoire suggests that the Bulbul is an “open‐ended learner”, and that the pattern of syllables that make up motifs allows the formation of a wide variety of motifs in each population. It is likely that this pattern exists in both males and females, as we found that both use complex vocalizations to communicate (Figure S1).

### 5.2 Syllable Similarities Between Populations

The various motifs that characterize each population are composed of syllables, which in contrast to motifs, are much more similar between populations. We defined 7–9 syllables in each population, and by applying two different methods to analyze syllable similarity, it appears that at least some of the syllables are used by all populations (Figure 6).

Studies on bird's vocal units have found distinct syllable types, many of which exhibit similarity within populations (i.e., between different individuals in a population) or between close populations, showing that the degree of syllable sharing decreases with geographic distance. For example, a study on male Rufous‐capped warblers has found a complex repertoire consisting of a finite number of syllables that different individuals recombine to produce a large number of song versions. In this case, they concluded that syllables, rather than whole songs, are the basic learned unit of the repertoire (Demko and Mennill 2019). Studies that have found syllable sharing, usually refer to neighboring populations or to neighboring individuals within the same population (McGregor and Krebs 1989; Baker et al. 2000; MacDougall‐Shackleton and MacDougall‐Shackleton 2001; Lachlan et al. 2018; Demko and Mennill 2019; Lee et al. 2019; Webb et al. 2021).

In a similar way, our findings suggest that individuals in the same population share syllables and recombine them in different motifs. Furthermore, our results imply that even geographically distant populations use the same syllables, as we found high similarity between syllables that are used in Jerusalem (a distant population) to those in the northern population (Figure S9). While syllable types are shared across populations, the way these syllables are combined into motifs differs, indicating that motif syntax is dialect‐specific, whereas the syllable inventory appears to be more shared across the species' populations (see Figure 4g,i,j).

It may be useful to consider two alternative hypotheses regarding the interrelations between syllables and the motifs they form: (a) Syllables may be more morphologically constrained and hence more genetically determined. This may lead them to being relatively limited in number and in form. If this is the case, syllable variation between populations should be attributed to physiological, morphological, and genetic differences between them. Consequently, we would expect to see syllables from different populations clustered together. (b) Vocal units' variation is due to cultural drift, whereby both syllables and motifs are learned. Thus, over time and distance, vocal units would be transmitted with slight changes due to copy errors. These continuous modifications would blur any distinct cluster between populations. This could be tested by analyzing and comparing syllables and motifs in different populations along a geographical path. Overall, if there is a pattern in which the similarity between syllables decreases with geographical distance, as many studies show, we would expect to see a significant difference between the northern populations and the Jerusalem population. Instead, we found that motifs (Figure 4) seem to form separate and distinct clusters even between close populations, whereas the syllables (Figure 6) are much less distinct and overlap across all populations. Therefore, we suggest that syllables are more structured and conserved, while motifs are a product of learning or invention and are more subject to changes or cultural transmission. However, in the presence of vocal learning, some aspects of the neural architecture underlying song production may be genetically determined. Our results are consistent with the idea that the observed population‐level differences in motifs likely reflect an interplay between innate (genetically influenced) and learned components. To disentangle these contributions and to determine whether Bulbuls' syllables are shared or simply exhibit random similarities, future studies involving experimental manipulations—such as cross‐fostering or playback experiments across populations—would be valuable, albeit challenging to implement under field conditions (As in Suzuki et al. 2016 or Bistel et al. 2025). If different syllable combinations and orders convey different meanings, it is likely that the presence of a specific syllable type is consistent and shared between individuals and populations, rather than being subject to random variations.

In summary, in this study we tested our pipeline on four Bulbul populations, providing a robust framework that allows processing large amounts of data with minimal manual intervention and to analyze the data efficiently while maintaining its acoustic complexity, providing a more accurate representation of the species' vocal behavior. Moreover, our methods can be further utilized to analyze hourly, daily, and monthly vocal activity, to explore trends or changes across seasons and years, and to identify patterns of vocal behavior. Our findings support our observations that Bulbuls use distinct motifs that form a diverse local repertoire. These motifs consist of base units (syllables), some of which appear to be shared by nearby and distant populations. These findings support the possibility that syllables are genetically constrained and potentially influenced by basic innate traits such as the size of the syrinx, the shape of the bill, or the tongue, whereas motifs are shaped by non‐innate features such as cultural transmission, learning ability, innovation, and more.

## Author Contributions

**Aya Marck:** writing – original draft (equal). **Oren Kolodny:** writing – original draft (equal). **Yoni Vortman:** writing – original draft (equal). **Rachel Ben‐Shlomo:** writing – original draft (equal). **Yizhar Lavner:** writing – original draft (equal).

## Conflicts of Interest

The authors declare no conflicts of interest.

## Supporting information


**Data S1:** ece372020‐sup‐0001‐Supinfo.docx.

## Data Availability

The analysis methods and datasets for this study can be found in GitHub, in the following link: https://github.com/BulbulNET?tab=repositories.
