# Supplementary material for: Building Complexity From Simplicity: A Songbird's Vocal Repertoire Varies Among Populations Despite Similarity of Syllables
Source: Ecol Evol. 2025 Sep 10;15(9):e72020. doi: 10.1002/ece3.72020 (PMC12422752; doi:10.1002/ece3.72020)
Supplement: Supplementary file 1 — Data S1: ece372020‐sup‐0001‐Supinfo.docx. [file ECE3-15-e72020-s001.docx]

# Supplementary

**Supplementary 1**

Focal recordings – At the beginning of the study, we conducted focal recordings under the assumption that it would be relatively straightforward to record individuals marked with colored rings within their natural population. In total, 361 recordings were collected at Agmon and Jerusalem, of which 42 contained identifiable markings corresponding to 26 individuals. DNA was successfully extracted from nine of these identified individuals. Recordings were made in the mornings between early August and late October 2020, from a distance of less than 10 meters. For each recording, we documented a standardized protocol, including ring colors, the number of individuals, observed behaviors, and other relevant details. The data collected was not sufficient to characterize a repertoire, but it certainly provided behavioral information, such as these recordings - of complex vocalizations from both males and females.


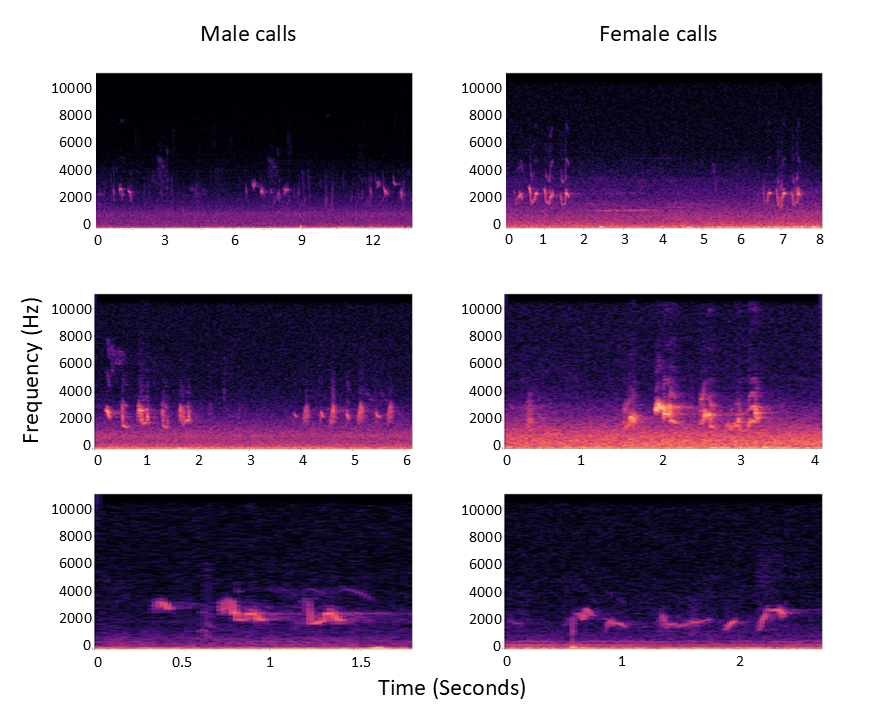


**Supplementary Figure S1**- Spectrograms of different male calls (on the left column) and female calls (on the right column). These recordings were made using a manual recorder (Tascam recorder and Sennheiser ME67 long shotgun), when it was possible to identify the Bulbul by the colored rings on its feet. Following to the individual identification, it was possible to cross-check this recording with the information regarding the individual's sex (DNA samples were taken from most of the marked individuals). Both male and female produce complex vocalizations.

**Supplementary 2**

motif boundaries demarcation

The input to BulbulNet detection module is a sequence of mel-spectrograms each calculated from segments of one second with a hop size of 0.5 second. The output of the deep learning model is therefore a binary sequence of Bulbul (“1”) or not Bulbul (“0”) decisions. Therefore, the identifications were not edited exactly according to the beginning and end of the vocalization. To obtain isolated and complete motifs a unification of overlapping segments recognized as Bulbul vocalizations was carried out. For a more accurate demarcation we used a simple algorithm which is based on isolated spoken motif detection (Rabiner and Sambur, 1975). For each motif’s signal the short-time energy is computed (with frame length of 10 ms and 50% overlap). The energy is normalized to [0,1] and two thresholds are calculated:

$${Th}_{perm}= max(p_{20}, Th), {Th}_{cons}= min(p_{20}, Th)$$

where ${Th}_{perm}$ and ${Th}_{cons}$are a permissive and a conservative threshold, respectively, $p_{20}$ is the 20^th^ percentile of the signal energy, and $Th$ is a predefined initial threshold. A start time is set as the first point in the signal (moving from left to right) for which the signal energy is greater than ${Th}_{cons}$. Then by moving backwards (from right to left), the final start time is defined as the point whose energy is lower then ${Th}_{perm}$. The end time of the motif is found in a similar way. The demarcated signal was considered as an isolated motif and was extracted for further analysis.

**Supplementary 3**


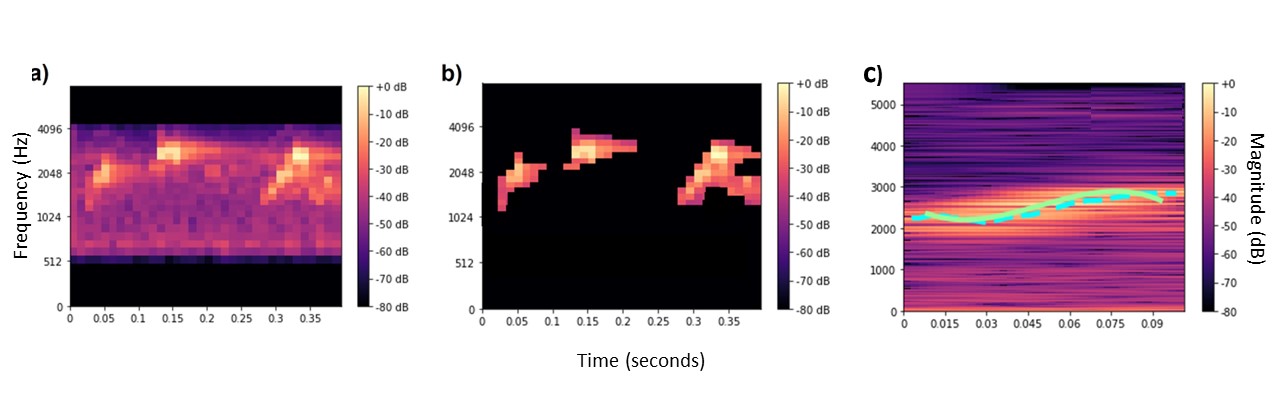


**Supplementary Figure S**3 – a) Mel-spectrogram of the motif '*tu ti tuyu*' b) The same signal after median clipping. The result is a much clearer image of the signal.. c) A spectrogram of a syllable. The fundamental frequency contour estimation marked in blue-cyan, and the 3rd degree Legendre polynomial model marked in light green.

**Supplementary 4**


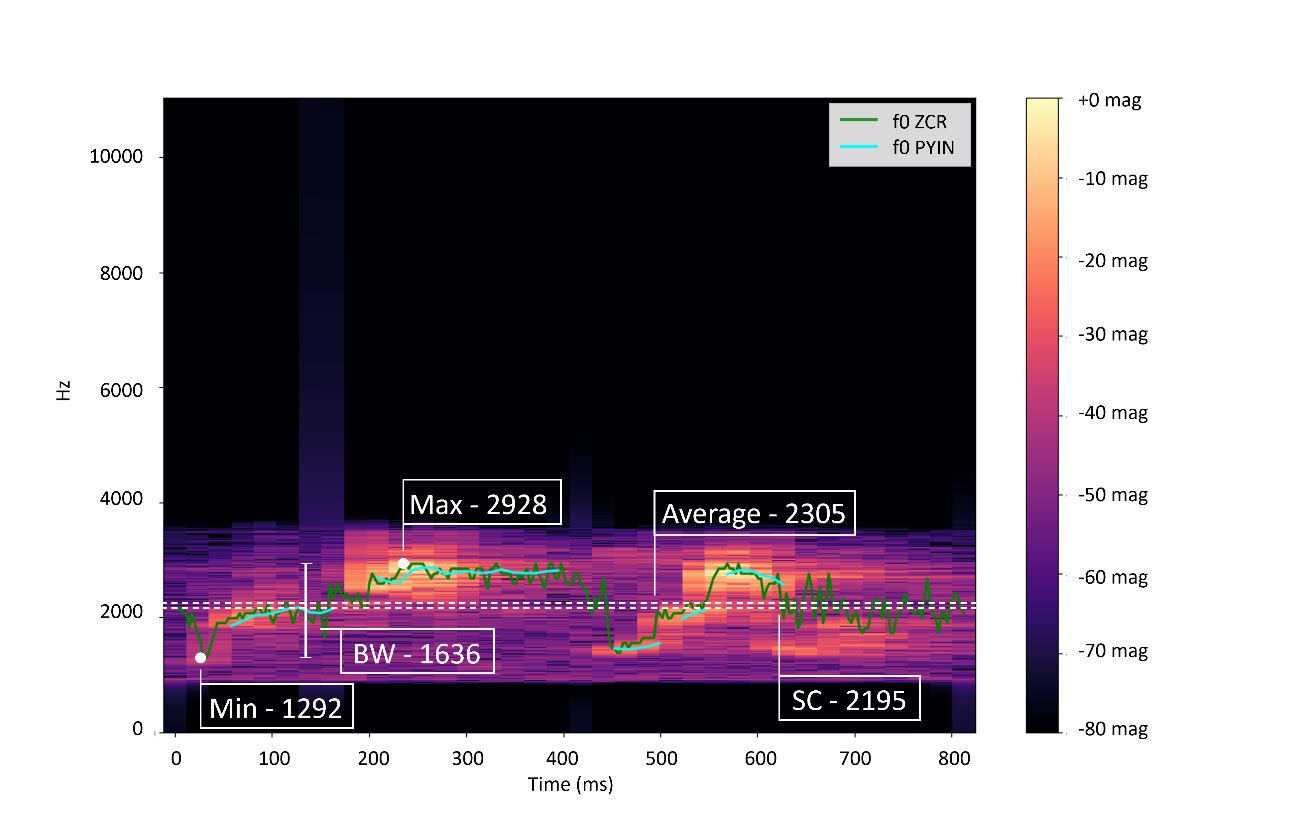


**Supplementary Figure S4** – An illustration of the parameter's extraction process, where the signal represented using a mel- spectrogram of a motif with three syllables, after the cleaning and cutting process (the initial motif was 1.1 second long with intervals between the syllables and was processed to a 0.8-second continuous motif). The motif segments, detected using the deep neural network model (see Methods, 3.1), were filtered using a bandpass filter (700–3900 Hz), where the fundamental frequency of nearly all bulbul vocalizations resides. The fundamental frequency was then estimated using a pitch detection algorithm (PYIN or ZCR, Marck et al., 2022). Parameters such as maximum fundamental frequency (Max), minimum fundamental frequency (Min), the bandwidth of the signal (BW), average fundamental frequency (Avg) and spectral centroid (SC) were automatically extracted from the fundamental frequency estimation (blue-cyan for PYIN and dark green for ZCR), except for the spectral centroid which was calculated directly from the spectrogram).

**Supplementary 5**

| **Source** | **Species** | **Successful amplification** | **Annealing temperature** | **Primer sequence (5'–3')** | **Locus** |
| --- | --- | --- | --- | --- | --- |
|  |  |  |  | Sex typing | |
| Griffiths et al. (1998) | Many birds' orders | V | 48 | TCTGCATCGCTAAATCCTTT | P2 |
|  |  |  |  |  |  |
|  |  | V |  | CTCCCAAGGATGAGRAAYTG | P8 |
|  |  |  |  | Microsatellite analysis | |
| Wu et al. (2011) | *Hemixos castanonotus* | - |  | TCTGTCTGTCTGTCTGTGGGTG | Heca4 |
|  |  |  |  | ATCAGGAGGTGGAGTGAAGC |  |
| Wu et al. (2011) | *Hemixos castanonotus* | - |  | CAGGTGAAGTGACATGGCAG | Heca5 |
|  |  |  |  | CTGGAACATTCCCTTCTCCC |  |
| Wu et al. (2011) | *Hemixos castanonotus* | V | 64 | ATCTGGGTGGGTGCGTGTCT | Heca8 |
|  |  |  |  | CCATCAGGAGGTGGAGTGAA |  |
| Wu et al. (2011) | *Hemixos castanonotus* | monomorphic | 56 | GCAGGGTTCTTCTTGGCAAT | Heca12 |
|  |  |  |  | CGCTTTCCACCAGAGCACAG |  |
| Wu et al. (2011) | *Hemixos castanonotus* | - |  | GGACACCTGCTCTGCCTTGC | Heca14 |
|  |  |  |  | GACAGATGTGCCCTTGTTGC |  |
| Lokugalappatti et al. (2008) | *Phyllastrephus flavostriatus* | V | 60 | GTGCAGTTTCGGTTGTTTCCC | Pf135 |
|  |  |  |  | CCATGGTACTGTTAGAGATCGGTATC |  |
| Dawson et al. (2015) | *Pycnonotus xanthopygos* | V (Z chromosome) | 56 | AAAACACCTTGTAATTTAAAACTGG | Z-037 |
|  |  |  |  | CATAGATACATATCAATACAGCACATTC |  |
| Dawson et al. (2015) | *Pycnonotus xanthopygos* | V (Z chromosome) | 56 | AAAAGTCTTTCTGGACTGTGCT | Z-040 |
|  |  |  |  | AAAATACAACAGACATAGGCATACA |  |
| Lokugalappatti et al. (2008) | *Phyllastrephus flavostriatus* | V | 56 | GCAGCGTCTAACCAATAACTCCTG | Pf151 |
|  |  |  |  | CTGATTAATACAGTGACTTGGCTTTCACC |  |
| Lokugalappatti et al. (2008) | *Phyllastrephus flavostriatus* | V | 64 | GCCCCATCTTTCCTTACTGGG | Pf152 |
|  |  |  |  | GCCAGATCATAACACTAACAGGCC |  |
| Lokugalappatti et al. (2008) | *Phyllastrephus flavostriatus* | V | 64 | GGTGTGCAGAATTTGGCTGC | Pf177 |
|  |  |  |  | CTGCTGATCTTCCAGCCCTTC |  |
| Page et al. (2014) | *Alophoixus pallidus* | - |  | AGAGGAAGAATGGGAGTGGG | PpMs4 |
|  |  |  |  | AACTGCATCACCCTTGGAGG |  |

**Supplementary Table S5** - List of the different primers used, selected according to the literature; only two of them were previously tested on this species. We successfully amplified (marked as V) fragments from 10 out of 14 tested primer pairs (including sex typing), two of them were Z-linked and one was monomorphic. As a result, 5 primers were used for microsatellite analysis.

**Supplementary 6**

|  | **Agmon** | **Shear Yashuv** | **Yiftah** | **Jerusalem** |
| --- | --- | --- | --- | --- |
| **Agmon** | 0.000 |  |  |  |
| **Shear Yashuv** | 0.005 | 0.000 |  |  |
| **Yiftah** | 0.014 | 0.041 | 0.000 |  |
| **Jerusalem** | 0.084 | 0.074 | 0.041 | 0.000 |

**Supplementary Table S6** – genetic distance (D) among populations, calculated with Nei Index.

**Supplementary 7**

| **Population** | **N** |  | **Na** | **Ho** | **He** | **uHe** | ***F*** |
| --- | --- | --- | --- | --- | --- | --- | --- |
| Agmon | 23 | Mean | 4.400 | 0.346 | 0.525 | 0.538 | 0.239 |
|  |  | SE | 1.030 | 0.054 | 0.097 | 0.100 | 0.161 |
| She’ar Yashuv | 13 | Mean | 4.600 | 0.600 | 0.621 | 0.646 | 0.020 |
|  |  | SE | 1.122 | 0.089 | 0.071 | 0.074 | 0.117 |
| Yiftah | 12 | Mean | 3.800 | 0.567 | 0.612 | 0.638 | 0.028 |
|  |  | SE | 0.663 | 0.103 | 0.064 | 0.067 | 0.180 |
| Jerusalem | 17 | Mean | 4.400 | 0.437 | 0.627 | 0.646 | 0.278 |
|  |  | SE | 0.927 | 0.074 | 0.071 | 0.073 | 0.117 |
| **Total** | **65** | **Mean** | 4.300 | **0.487** | **0.596** | 0.617 | **0.141** |
|  |  | **SE** | 0.442 | **0.044** | **0.037** | 0.038 | **0.072** |

**Supplementary Table S7** - A summary of genetic diversity parameters. N - number of individuals, Na - number of different alleles, Ho - observed heterozygosity in population, He - expected heterozygosity, uHe - unbiased expected heterozygosity (2N / (2N-1)) * He and F - Fixation Index = (He - Ho) / He = 1 - (Ho / He).

**Supplementary 8**

We applied PERMANOVA to test the null hypothesis that the centroids of the populations motifs in the ordination space do not differ significantly between groups. PERMANOVA revealed significant differences in motif structure between populations (F = 0.046, p = 0.0002). Although the pseudo-F value is modest, the very low p-value indicates that the observed grouping structure is highly unlikely to have occurred by chance. This suggests that the multivariate differences between populations, while subtle, are consistent and statistically meaningful. Taking together with the t-SNE visual representation (Fig 4) it reveals an internal structure within each population, as the different motifs (sub-clusters) are distinct but do not cluster tightly together to one center of each population. This within-group heterogeneity reflects biologically meaningful variation as we expected, where the motifs share the same potential phonemic space, and there is a clear distinction between them. This test was followed by post hoc tests for each pair of groups, all of which were found to be significantly different.

**Supplementary 9**


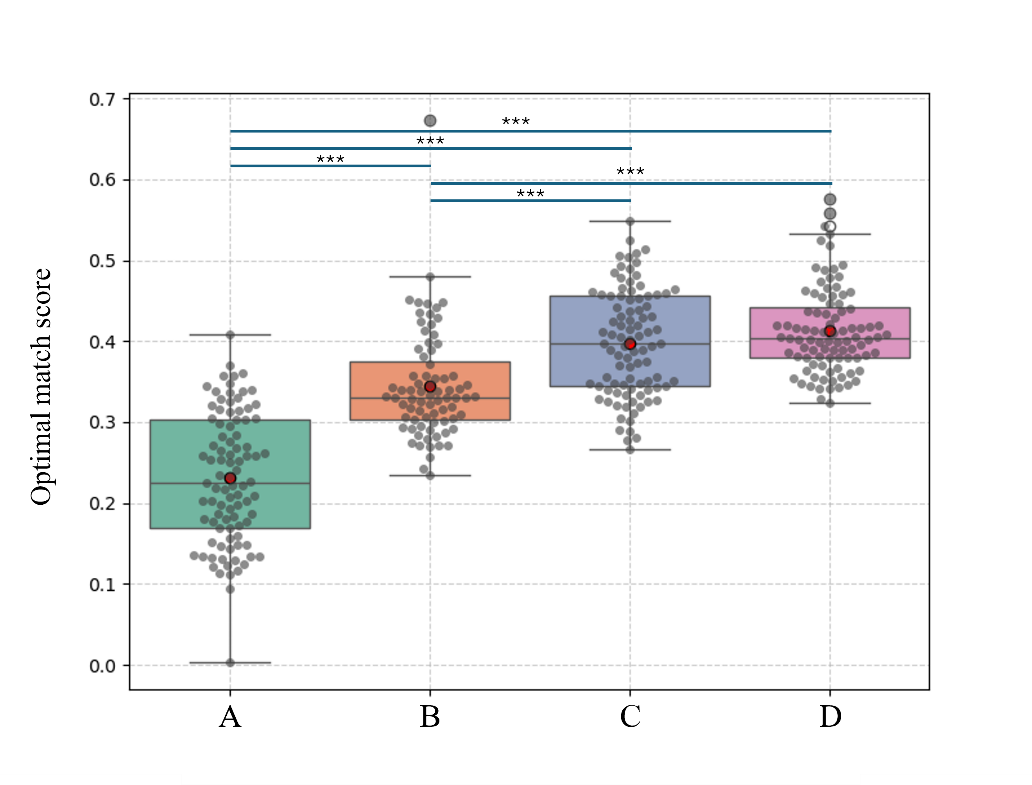


**Supplementary Figure S9**– A summary of the average score of optimal match between- A) syllables from the same population that we identified as the same (n = 92, optimal match - 0.242). B) syllables that we identify as the same among different motifs in different population (n = 76, optimal match - 0.345). C) different syllables within the same population (n = 92, optimal match - 0.398). D) different syllables among different populations (n = 92, optimal match - 0.413). The score was calculated between all pairwise syllables, using DTW. Values are between [0,1] where low values indicate high similarity. Same syllables in different populations (B) are more similar than other syllables within population (C) and between populations (D). Kruskal-Wallis test followed by Dunn’s post hoc test indicate that groups are significanly diffarent from each other (***p<0.001), with the exeption of groups C and D that are not significantly different (p = 0.870).

**Supplementary 10**

| **Feature** | **location** | **mean** | **median** | **std** | **p value** |
| --- | --- | --- | --- | --- | --- |
| **Maximum fundamental frequency** | Agmon | 3077 | 3100 | 268 | **Agmon - Jerusalem < 0.001**  Agmon – She'ar Yashuv - ns  Agmon - Yiftah - ns  **Jerusalem – She'ar Yashuv < 0.001**  **Jerusalem - Yiftah < 0.001**  She'ar Yashuv – Yiftah - ns |
|  | **Jerusalem *** | 2944 | 22928 | 268 |  |
|  | She'ar Yashuv | 3071 | 3100 | 259 |  |
|  | Yiftah | 3152 | 3186 | 323 |  |
|  |  |  |  |  |  |
|  |  |  |  |  |  |
| **Minimum fundamental frequency** | Agmon | 1756 | 1722 | 266 | **Agmon - Jerusalem < 0.001**  Agmon – She'ar Yashuv - ns  **Agmon - Yiftah < 0.001**  **Jerusalem – She'ar Yashuv < 0.001**  **Jerusalem - Yiftah < 0.001**  **She'ar Yashuv - Yiftah < 0.001** |
|  | **Jerusalem *** | 1603 | 1550 | 236 |  |
|  | She'ar Yashuv | 1794 | 1722 | 251 |  |
|  | **Yiftah *** | 1718 | 1722 | 247 |  |
|  |  |  |  |  |  |
|  |  |  |  |  |  |
| **Average fundamental frequency** | Agmon | 2465 | 2470 | 215 | **Agmon - Jerusalem < 0.001**  Agmon – She'ar Yashuv - ns  **Agmon - Yiftah < 0.001**  **Jerusalem – She'ar Yashuv < 0.001**  **Jerusalem - Yiftah < 0.001**  **She'ar Yashuv - Yiftah < 0.001** |
|  | **Jerusalem *** | 2272 | 2249 | 209 |  |
|  | She'ar Yashuv | 2457 | 2466 | 205 |  |
|  | **Yiftah *** | 2439 | 2443 | 234 |  |
|  |  |  |  |  |  |
|  |  |  |  |  |  |
| **Bandwidth** | Agmon | 1320 | 1378 | 376 | Agmon - Jerusalem - ns  Agmon – She'ar Yashuv - ns  **Agmon - Yiftah < 0.001**  Jerusalem – She'ar Yashuv - ns  **Jerusalem - Yiftah < 0.001**  **She'ar Yashuv - Yiftah < 0.001** |
|  | Jerusalem | 1340 | 1378 | 356 |  |
|  | She'ar Yashuv | 1277 | 1291 | 357 |  |
|  | **Yiftah *** | 1432 | 1464 | 288 |  |
|  |  |  |  |  |  |
|  |  |  |  |  |  |
| **Mean spectral centroid** | Agmon | 2399 | 2395 | 165 | **Agmon - Jerusalem < 0.001**  Agmon – She'ar Yashuv - ns  Agmon - Yiftah - ns  **Jerusalem – She'ar Yashuv < 0.001**  **Jerusalem - Yiftah < 0.001**  **She'ar Yashuv - Yiftah < 0.002** |
|  | **Jerusalem *** | 2268 | 2247 | 152 |  |
|  | She'ar Yashuv | 2398 | 2398 | 158 |  |
|  | Yiftah | 2398 | 2404 | 181 |  |
|  |  |  |  |  |  |
|  |  |  |  |  |  |

**Supplementary Table S10** – Mean, median and standard deviation of the acoustic features histograms presented in figure 4 together with the results of Kruskal–Wallis test and Dunn post-hoc test. Dunn test showed that the vocalizations of Yiftah population is significantly different from all other populations in 3 out of 5 features, and similarly Jerusalem population is significantly different from the others in 4 out of 5 features.
